# Skin-Derived TSLP Triggers Progression from Epidermal-Barrier Defects to Asthma

**DOI:** 10.1371/journal.pbio.1000067

**Published:** 2009-05-19

**Authors:** Shadmehr Demehri, Mitsuru Morimoto, Michael J. Holtzman, Raphael Kopan

**Affiliations:** 1 Department of Developmental Biology and Division of Dermatology, Washington University School of Medicine, Saint Louis, Missouri, United States of America; 2 Department of Medicine, Washington University School of Medicine, Saint Louis, Missouri, United States of America; MD Anderson Cancer Center, United States of America

## Abstract

Asthma is a common allergic lung disease frequently affecting individuals with a prior history of eczema/atopic dermatitis (AD); however, the mechanism underlying the progression from AD to asthma (the so-called “atopic march”) is unclear. Here we show that, like humans with AD, mice with skin-barrier defects develop AD-like skin inflammation and are susceptible to allergic asthma. Furthermore, we show that thymic stromal lymphopoietin (TSLP), overexpressed by skin keratinocytes, is the systemic driver of this bronchial hyper-responsiveness. As an AD-like model, we used mice with keratinocyte-specific deletion of RBP-j that sustained high systemic levels of TSLP. Antigen-induced allergic challenge to the lung airways of RBP-j–deficient animals resulted in a severe asthmatic phenotype not seen in similarly treated wild-type littermates. Elimination of TSLP signaling in these animals blocked the atopic march, demonstrating that high serum TSLP levels were required to sensitize the lung to allergic inflammation. Furthermore, we analyzed outbred *K14-TSLP^tg^* mice that maintained high systemic levels of TSLP without developing any skin pathology. Importantly, epidermal-derived TSLP was sufficient to trigger the atopic march, sensitizing the lung airways to inhaled allergens in the absence of epicutaneous sensitization. Based on these findings, we propose that in addition to early treatment of the primary skin-barrier defects, selective inhibition of systemic TSLP may be the key to blocking the development of asthma in AD patients.

## Abstract 2

A skin-derived cytokine with high systemic availability provides a mechanistic explanation for atopic march and highlights a potential therapeutic target for preventing the development of asthma among people with atopic dermatitis.

## Author Summary

Eczema (atopic dermatitis) is a common allergic skin inflammation that has a particularly high prevalence among children. Importantly, a large proportion of people suffering from eczema go on to develop asthma later in life. Although the susceptibility of eczema patients to asthma is well documented, the mechanism that mediates “atopic march”—the progression from eczema to asthma—is unclear. We used genetic engineering to generate mice with chronic skin-barrier defects and a subsequent eczema-like disorder. With these mice, we were able to investigate how skin-specific defects predisposed the lungs to allergic asthma. We identified thymic stromal lymphopoietin (TSLP), a cytokine that is secreted by barrier-defective skin into the systemic circulation, as the agent sensitizing the lung to allergens. We demonstrated that high systemic levels of skin-derived TSLP were both required and sufficient to render lung airways hypersensitive to allergens. Thus, these data suggest that early treatment of skin-barrier defects to prevent TSLP overexpression, and systemic inhibition of TSLP, may be crucial in preventing the progression from eczema to asthma.

## Introduction

Allergic asthma is a chronic lung disease characterized by T-helper2 (Th2)–mediated inflammation and airway obstruction in response to allergen exposure [1]. Asthma is an increasingly common disorder affecting more than 300 million individuals around the world [2]. Atopic dermatitis (AD) is another prevalent allergic disease, and 17% of children in the United States suffer from this disorder [3]. In comparison to its 4–8% prevalence in the general population, asthma develops in up to 70% of patients with history of severe AD, a phenomenon referred to as “atopic march” [3]. It may be possible to prevent asthma in these at-risk individuals by early diagnosis of AD and blockage of the atopic march. To achieve this, it is critical that we understand the mechanism triggering the development of asthma in AD patients.

A survey of the literature has identified several possible mechanisms underlying the clinical link between AD and asthma. These include: (a) a systemic immune system disorder leading to excessive Th2 response at epithelial surfaces exposed to allergens [4], (b) a barrier defect shared by both skin and lung epithelia that leads to overstimulation of the immune cells by invading allergens [5], or (c) systemic consequences of a skin-specific barrier defect causing immune cells to mount an allergic inflammation at any allergen-exposed epithelial surface [6]. Epidemiological data supporting the third hypothesis include the observation that AD tends to be the first manifestation of the atopic march [3]. Another supportive observation relates to filaggrin, a skin cornified envelope protein that is absent from the lung epithelia [7],[8]. Although controversial [9], it seems that AD patients with filaggrin loss-of-function mutations exhibit increased incidence of asthma [10]–[12]. This is consistent with the third hypothesis, that loss of an epidermal-specific barrier protein can trigger systemic atopy in humans. Mechanistically, it is suspected that epicutaneous sensitization with allergens underlies the development of airway hyperreactivity in mice [13] and in humans with filaggrin mutations [10],[11]. However, it is unclear whether intrinsic skin-barrier defects can trigger asthma in the absence of any epicutaneous sensitization. If epicutaneous exposure is not required, it will imply that systemic factor(s) produced by AD skin may be involved in sensitizing the bronchial epithelia. Such factors will be important as therapeutic targets in preventing asthma.

To address this question, we studied mice lacking Notch signaling in the skin. Skin keratinocytes are organized in highly interconnected basal, spinous, granular, and cornified layers, forming an elaborate barrier protecting the organism from the outside environment. One of the major molecular regulators of this structure is Notch signaling [14]–[16]. Notch is a transmembrane receptor interacting with ligands expressed on the surface of neighboring keratinocytes [16],[17]. Upon activation, sequential proteolysis of Notch releases its intracellular domain, which then translocates into the nucleus, binds to RBP-j (the DNA-binding partner of Notch), and activates downstream targets [17]. Keratinocyte-specific deletion of Notch signaling pathway components impairs epidermal differentiation, resulting in skin-barrier defects [15],[16]. We have recently shown that Notch signaling loss in the skin also triggers a severe neonatal B-lymphoproliferative disorder (B-LPD; [15]). This systemic disease is directly caused by elevated levels of thymic stromal lymphopoietin (TSLP) released into the circulation by Notch-deficient keratinocytes failing to differentiate [15]. TSLP, a general biomarker for skin-barrier defects [15], is an interleukin-7 (IL-7)–like cytokine produced by epithelial cells and is implicated in the pathogenesis of both AD and asthma [18]–[22]. TSLP expression is sustained as long as barrier defects persist [15]. Thus, the potentially high systemic availability of skin-derived TSLP and its central role in promoting asthma bring up the possibility that TSLP may be the factor predisposing AD patients to asthma.

Identifying a clear mechanism for the atopic march in the complex network of genetic, immunological, and environmental factors that contribute to AD has been a challenge to the field and has resulted in much debate as to the right therapeutic approach for preventing asthma [6]. To determine the mechanisms underlying the progression from AD to asthma, we first generated animals that lacked Notch signaling in a portion of their skin surfaces by embryonic removal of RBP-j from keratinocytes using the *Msx2-Cre* transgene (*Msx2-Cre/+;RBP-j^flox/flox^* or RBP-jCKO). *Msx2-Cre* is ectopically expressed at embryonic day 9.5 (E9.5) in clusters of ectodermal cells, resulting in a chimeric pattern of *RBP-j* deletion in the skin [23]. The presence of unaffected skin surfaces allowed RBP-jCKO animals to live for approximately 100 days on average with a few surviving up to one year (Figure S1) [15],[16], presenting a model in which to determine whether a defective skin-barrier could cause AD-like symptoms and render susceptibility to asthma in the absence of epicutaneous sensitization. We found that defective skin-barrier function in adult RBP-j–deficient animals caused the development of an AD-like allergic inflammation and a subsequent susceptibility to asthma. To determine whether TSLP is required for this susceptibility, we deleted the TSLP receptor in RBP-jCKO mice and showed that this genetic manipulation blocked the development of asthma in animals with persistent AD-like pathology and inflammation. To ask if TSLP overexpression by skin keratinocytes is sufficient, we used outbred transgenic mice overexpressing TSLP in epidermal keratinocytes and showed that epidermal-derived TSLP was sufficient to confer a severe asthmatic phenotype even in the absence of any skin defect. These findings establish that high systemic availability of TSLP [15] can sensitize the lung to allergens, and provide a novel molecular mechanism for the atopic march. Serum TSLP is thus an important potential therapeutic target in preventing asthma in AD patients.

## Results

### RBP-jCKO Mice Develop an AD-Like Skin Phenotype

**Figure 1:**
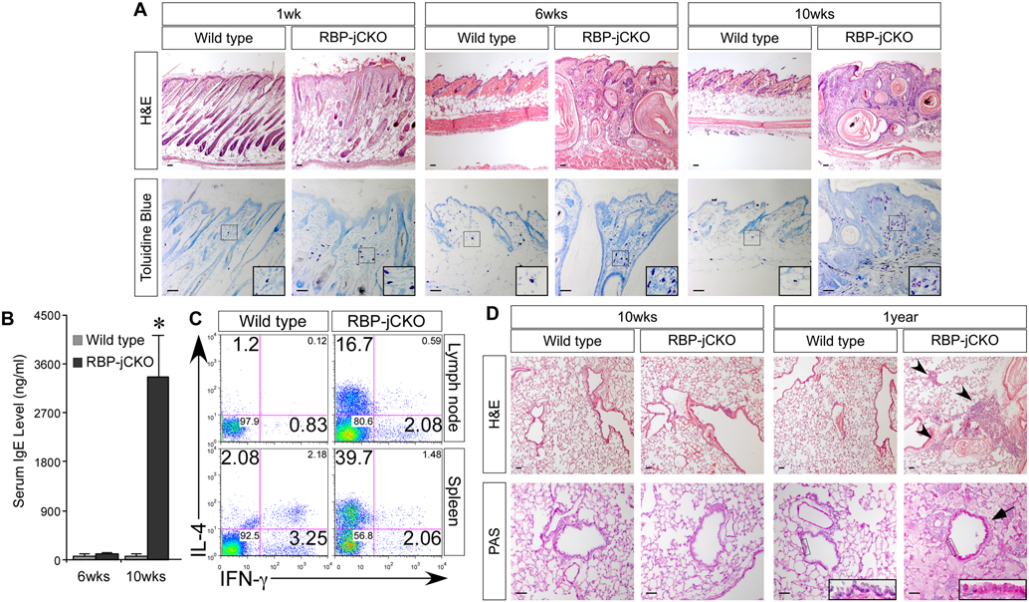
RBP-jCKO mice lacking RBP-j (and thus, notch signaling) in skin keratinocytes develop a progressive, AD-like disease culminating in aged animals with lung inflammation. (A) H&E and Toluidine blue staining of the skin documents severe AD-like changes in RBP-j–deficient mice, including epidermal hyperkeratosis, parakeratosis, acanthosis, skin inflammation, and increased number of dermal mast cells (insets) detectable as early as 1 wk after birth, which worsen as RBP-jCKO animals age (6 wk and 10 wk). Note that the keratin cysts formed in the dermis of RBP-jCKO mice are due to the destruction of RBP-j–deficient hair follicles [42]. (B) The progression of skin inflammation is reflected in RBP-jCKO serum IgE levels, which are dramatically elevated by 10 wk of age (10 wk; *n* = 4 for each group; *p<0.01). (C) Intracellular cytokine staining of CD4^+^ T cells isolated from lymph nodes (mixture of inguinal and axillary) or spleen shows a robust interleukin (IL)-4-positive and interferon (IFN)-γ-negative Th2 cell presence in 10-wk-old RBP-jCKO animals, indicating the development of a full-blown AD-like allergic inflammation. Percentage of cells in each quadrant is included (representative data are presented). (D) H&E and periodic acid-Schiff (PAS) staining of the lung do not show any signs of inflammation in 10-wk-old RBP-jCKO animals; however, at 52 wk (1 y), the mutant mice develop lung inflammation. The pathology of RBP-jCKO lung includes immune cell infiltration around the airways and blood vessels (arrowheads), goblet cell hyperplasia in the airways (arrow; insets), and airway remodeling (representative pictures are presented; scale bar: 50 µm).

The ablation of Notch signaling in skin keratinocytes by removing RBP-j severely impairs the differentiation of basal layer keratinocytes and maintenance of upper spinous and granular cell layers [15],[16]. Such a defect in epidermal stratification leads to an aberrant skin-barrier function signified by transepidermal water loss and penetration of dye through the defective barrier in RBP-j–deficient mice at birth [15],[16]. After birth, the persistence of barrier defects in RBP-j–deficient skin is evident by the presence of reactive epidermal hyperplasia, TSLP overexpression, and up-regulation of antimicrobial peptides (Figure S2 and Table S1) [15],[16],[24],[25]. Defective skin-barrier function has been suggested to be a hallmark of AD [6],[7]. In agreement with this notion, the impaired skin-barrier function in RBP-jCKO mice initiated an inflammatory cascade culminating in the development of an AD-like skin phenotype. The hyperplastic epidermis, acanthosis, hyperkeratosis, parakeratosis, and mast cell infiltration were evident in RBP-jCKO skin as early as 1 wk after birth, followed by dramatic dermal mast cell accumulation, serum IgE elevation, and systemic Th2 cell expansion in adult RBP-j–deficient animals (Figure 1A–1C). Therefore, adult RBP-jCKO mice resemble humans with AD to a degree that allows us to examine the systemic consequences of an allergic inflammation in the skin.

### AD-Like Skin Disease Predisposes RBP-jCKO Animals to Allergic Asthma

**Figure 2:**
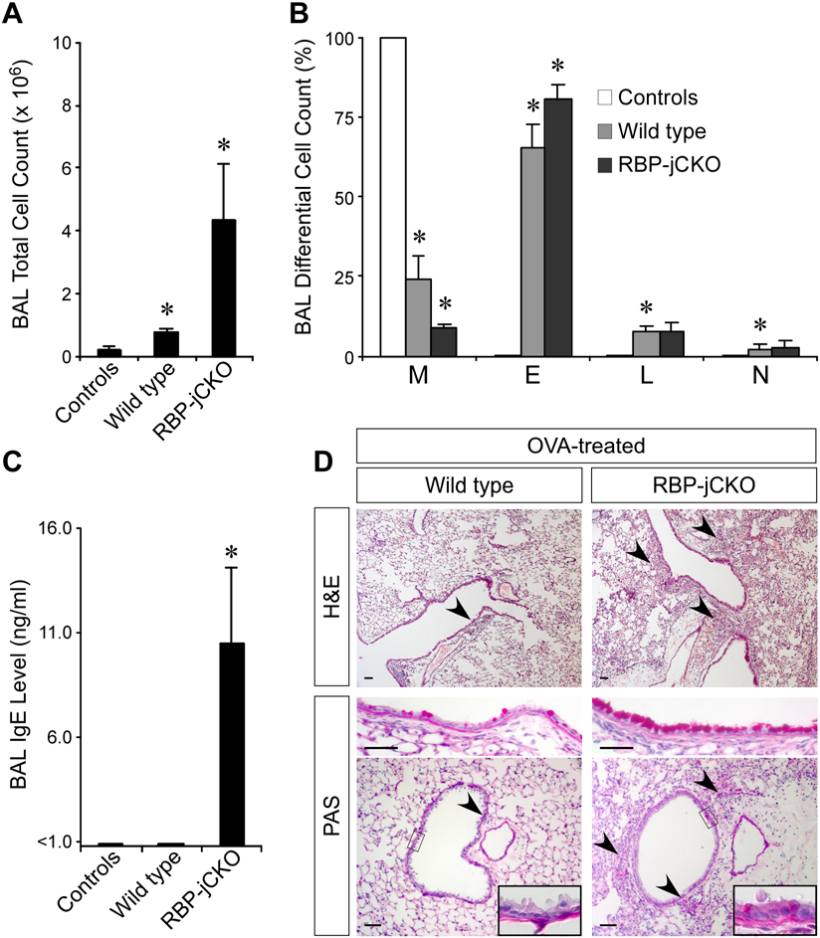
Five- to seven-wk-old RBP-jCKO mice develop a severe allergic lung inflammation in an OVA-induced murine model of asthma. (A and B) There are more leukocytes (A) and a higher percentage of eosinophils (B) present in BAL fluid collected from OVA-treated RBP-jCKO mice in comparison to their wild-type littermates (*Msx2-Cre/+;RBP-j^flox/+^*, *RBP-j^flox/flox^*, *RBP-j^flox/+^*), indicating a severe airway inflammation in these mutant animals (M, macrophages; E, eosinophils; L, lymphocytes; N, neutrophils). (C) IgE reaches detectable levels only in BAL fluid from RBP-j–deficient mice, further indicating the high intensity of OVA-induced allergic inflammation in the RBP-jCKO lung. (D) Comparing lung histology of RBP-jCKO with wild-type littermates sensitized and challenged with OVA shows a significantly more intense lung inflammation in RBP-jCKO mice. H&E and PAS staining shows greater immune cell infiltration around the airways and blood vessels (arrowheads), increased airway remodeling, goblet cell hyperplasia (insets), and mucus overproduction in the mutant lung (representative pictures are presented; scale bar: 50 µm). “Controls” refers to RBP-jCKO and wild-type animals treated with PBS instead of OVA (*n* = 4 for each group; *p<0.05, comparing the adjacent groups). These data are confirmed in additional independent experiments.

The lungs of 10-wk-old mice with RBP-j–deficient skins were normal and did not show any sign of allergic inflammation under standard housing conditions; however, the longest-living mutant animals (52 wk) did develop spontaneous lung inflammation (Figure 1D). To determine whether this phenotype was a true indicator of increased susceptibility to an asthmatic phenotype, we used an ovalbumin (OVA)-induced model of allergic inflammation with 5- to 7-wk-old RBP-jCKO mice. This protocol faithfully mimics the development of asthma in humans [18]. OVA-treated RBP-jCKO animals developed a more severe lung inflammation compared with OVA-treated wild-type littermates (Figure 2). Although the wild-type animals tolerated the intranasal OVA challenge well, two out of ten OVA-treated RBP-jCKO mice died during this procedure following a period of severe labored breathing. In the surviving mutants, the number of bronchoalveolar lavage (BAL) leukocytes and the percentage of BAL eosinophils were significantly higher compared with those of the wild-type littermates (Figure 2A and 2B). The absolute number of eosinophils was approximately 7-fold higher in the mutant mice. In addition, IgE was detectable only in the BAL fluid of RBP-j–deficient animals (Figure 2C). Histology of the lungs from OVA-challenged RBP-jCKO and wild-type controls clearly confirmed the existence of severe airway inflammation in RBP-jCKO mice, including significant leukocyte infiltration around the airways and blood vessels, extensive goblet cell hyperplasia in the large airways, and distinct appearance of goblet cells in medium-sized airways (Figure 2D). Considering that *RBP-j* is not deleted in the lung (Figures S3 and S7), these data clearly show that the skin-barrier defect can serve as a primary risk factor for development of asthma in a normal lung.

### TSLP Signaling Is Required for the Atopic March in RBP-j–Deficient Animals

It is possible that atopic skin lesions are the essential components downstream of skin-barrier defects that initiate systemic atopy in RBP-jCKO mice [3]. If this hypothesis were true, Th2 cells generated at the site of inflamed skin would migrate to other sites, including lung mucosa, and release high levels of Th2-derived cytokines, which would sensitize the lung airways to allergic inflammation. This model, however, is challenged by findings that show that Th2 cells generated in mouse models of AD specifically home to skin [26]. In addition, it is unclear whether the presence of AD lesions is required for initiation of the atopic march [11]. Skin-barrier defects led to systemic TSLP elevation [15], which remained elevated in the serum of RBP-jCKO animals throughout life (Figure S2). On the basis of these observations and that localized TSLP overexpression in lung epithelium is capable of inducing asthma [18],[19],[21], we have articulated an alternative hypothesis: TSLP may be a systemic signal that sensitizes the animals to allergen exposure in the lung. In that case, high systemic levels of epidermal-derived TSLP should render RBP-jCKO animals susceptible to the asthmatic phenotype upon exposure to allergen.

**Figure 3:**
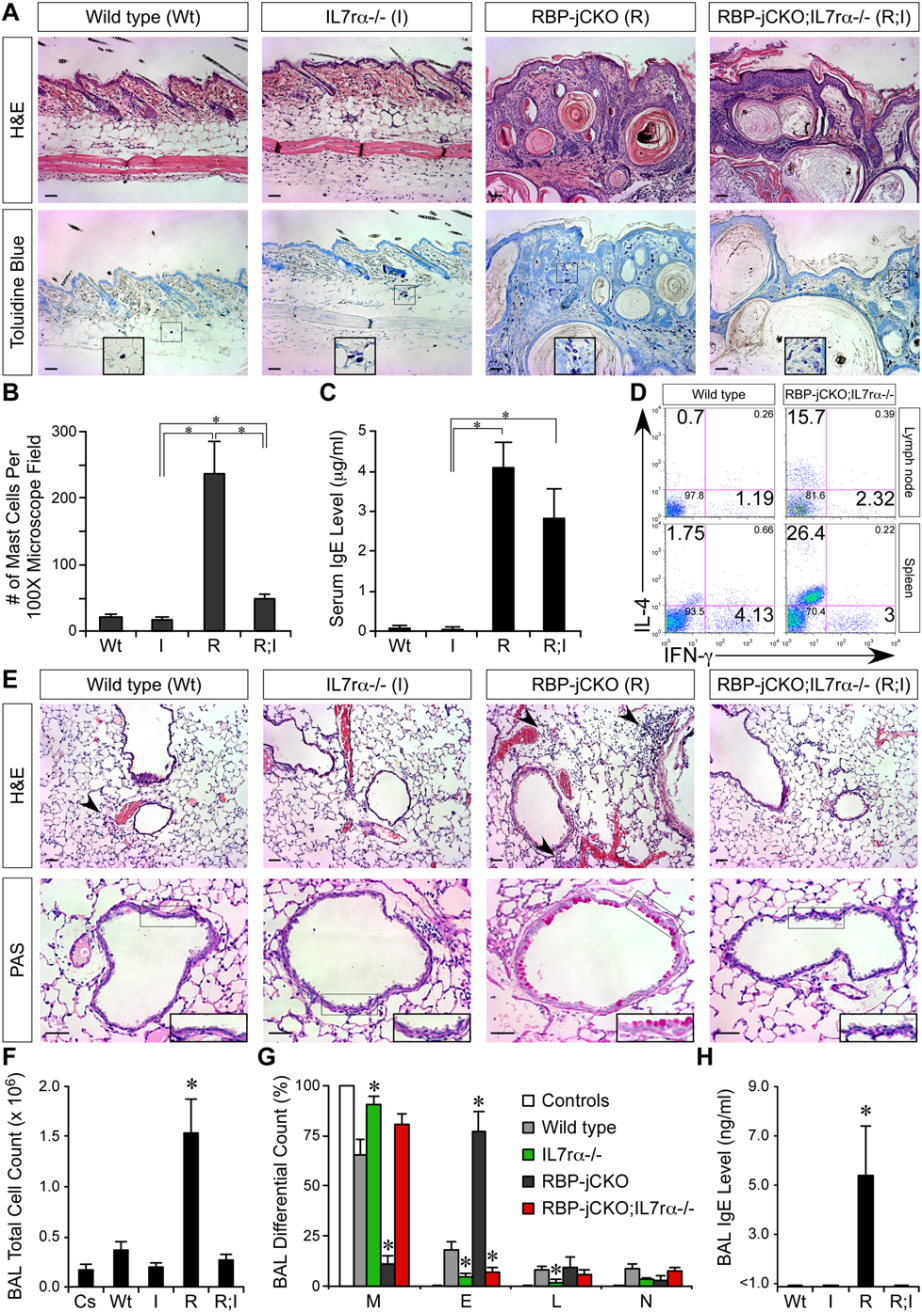
TSLP signaling blockade rescues asthmatic phenotype of RBP-jCKO mice. (A) H&E and Toluidine blue staining of the skin shows the persistence of epidermal hyperplasia, hyperkeratosis, parakeratosis, and acanthosis in 10-wk-old RBP-jCKO;IL7rα-/- skin. However, the local skin inflammation and number of dermal mast cells (insets) are markedly reduced in RBP-jCKO;IL7rα-/- skin (representative pictures are presented; scale bar: 50 µm [28]). (B) The quantitative analysis of mast cell infiltration in the dermis of RBP-jCKO;IL7rα-/- (R;I) shows that there is still more mast cell accumulation in RBP-jCKO;IL7rα-/- compared to wild-type (Wt) and IL7rα-/- (I) skin. The bar graphs show the average number of mast cells in ten random 100× microscope fields (*p<0.001). (C) Serum IgE levels of 10-wk-old RBP-jCKO;IL7rα-/- mice are highly elevated and are comparable to those of RBP-jCKO (*Msx2-Cre/+;RBP-j^flox/flox^;IL7r*α*+/-*, R) littermates (*p<0.01). (D) Intracellular cytokine staining shows a significant population of IL-4-producing Th2 cells in inguinal/axillary lymph nodes and spleen of 10-wk-old RBP-jCKO;IL7rα-/- animals (representative data are presented). (E) Comparing lung histology of 5- to 7-wk-old RBP-jCKO;IL7rα-/-, RBP-jCKO, IL7rα-/-, and wild-type littermates sensitized and challenged with OVA shows a complete reversal of the intense RBP-jCKO lung inflammation in RBP-jCKO;IL7rα-/- mice. H&E and PAS staining shows the muted inflammatory response around the airways and blood vessels (arrowheads), and lack of airway remodeling or goblet cell hyperplasia (insets) in RBP-jCKO;IL7rα-/- lung (representative pictures are presented; scale bar: 50 µm). (F and G) The average number of leukocytes (F) and percentage of eosinophils (G) in BAL fluid from OVA-treated RBP-jCKO;IL7rα-/- lung is lower than that of wild-type lung treated similarly, indicating that deletion of *IL7R*α rescues the allergic lung inflammation in RBP-jCKO mice (M, macrophages; E, eosinophils; L, lymphocytes; N, neutrophils). (H) IgE remains below detection levels in BAL fluid from OVA-treated RBP-jCKO;IL7rα-/- mice, further confirming that the intense OVA-induced allergic inflammation in RBP-jCKO lung is absent in RBP-jCKO;IL7rα-/- lung. To avoid death among RBP-jCKO cohort, all the animals in this study are challenged intranasally with OVA twice. Although the asthmatic response among RBP-jCKO;IL7rα-/-, IL7rα-/-, and wild-type groups is minimal, a severe response is seen in RBP-jCKO mice. “Controls” refers to RBP-jCKO;IL7rα-/-, RBP-jCKO, IL7rα-/-, and wild-type animals treated with PBS instead of OVA (*n* = 4 for each group; *p<0.01, compared to wild-type cohort). These data are confirmed in additional independent experiments.

To test this hypothesis we deleted the *IL7R*α subunit of TSLP receptor in RBP-jCKO animals (*Msx2-Cre/+*;*RBP-j^flox/flox^*;*IL7r*α-/-; or RBP-jCKO;IL7rα-/- [27]). This strategy was chosen because the *TSLPR* subunit of the TSLP receptor is linked to the *RBP-j* locus. The inhibition of TSLP reception reduced the severity of local AD-like inflammation in the skin of RBP-jCKO animals as judged by the reduction of dermal mast cells in RBP-jCKO;IL7rα-/- mice relative to their RBP-jCKO littermates [28]. However, a significant elevation in mast cell number was still detectable in RBP-jCKO;IL7rα-/- skin compared to wild type (Figure 3A and 3B). In addition, epidermal hyperplasia and TSLP overexpression that mark the presence of postnatal skin-barrier defects persisted in aged RBP-jCKO;IL7rα-/- mice (Figure 3A and Figure S4). Importantly, deletion of *IL7R*α did not affect the intensity of systemic Th2 response to allergic skin inflammation and serum IgE levels remained elevated in RBP-jCKO;IL7rα-/- mice (Figure 3C and 3D). This could be due to other cytokines/chemokines made by defective skin barrier in RBP-jCKO;IL7rα-/- animals [15], which together with exposure to allergens/pathogens through the compromised skin, sustain a robust systemic Th2 response even in the absence of TSLP reception [28]. Therefore, RBP-jCKO;IL7rα-/- animals provide a suitable system in which to determine if skin-barrier defects and AD-like skin inflammation including a systemic Th2 response with its consequences (e.g., elevated IgE) could confer susceptibility to asthma in the absence of TSLP signaling.

We challenged the lung airways of age- and sex-matched RBP-jCKO;IL7rα-/-, RBP-jCKO;IL7rα+/- (RBP-jCKO), IL7rα-/-, and wild-type littermates with OVA. To prevent RBP-jCKO lethality, all the animals in this experiment received intranasal OVA challenges only twice. Despite this reduced exposure, RBP-jCKO animals developed a severe asthmatic response, which was absent in RBP-jCKO;IL7rα-/- mice (Figure 3E–3H). Histological analysis could not detect goblet cell hyperplasia or significant inflammation around the airways and vasculature in RBP-jCKO;IL7rα-/- lungs (Figure 3E). Total white blood cell count and eosinophilia in BAL fluid from OVA-treated RBP-jCKO;IL7rα-/- mice was indistinguishable from IL7rα-/- littermates (Figure 3F and 3G). In addition, IgE was undetectable in BAL fluid from OVA-treated RBP-jCKO;IL7rα-/- mice (Figure 3H). These findings show that without a TSLP signal, no progression from allergic skin inflammation to asthma is observed, indicating that atopic march requires TSLP signals.

### Airway Hyper-Responsiveness in RBP-jCKO Mice Is Blocked by Inhibiting TSLP Signaling

**Figure 4:**
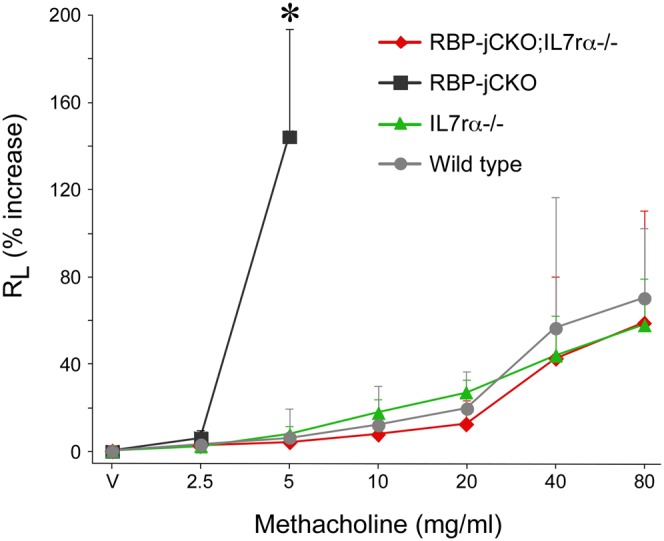
Severe airway hyper-responsiveness among OVA-treated RBP-jCKO mice is dependent on TSLP signaling. Age- and sex-matched RBP-jCKO;IL7rα-/-, RBP-jCKO, IL7rα-/-, and wild-type littermates are sensitized and challenged with OVA as in Figure 3. Airway reactivity at the basal state (vehicle alone; V) and in response to increasing doses of methacholine (2.5, 5, 10, 20, 40, and 80 mg/ml) is monitored in OVA-treated animals using total lung resistance (R_L_; *n* = 4 for each group). OVA-treated RBP-jCKO animals mount a severe airway hyper-responsiveness at 5 mg/ml of methacholine and do not tolerate methacholine doses less than or equal to 10 mg/ml, developing fatal labored breathing. OVA-treated RBP-jCKO;IL7rα-/-, IL7rα-/-, and wild-type mice, on the other hand, show moderate response only to the highest dose of methacholine (*p<0.01). Data are presented as mean + standard deviation of percent increase in total lung resistance compared to basal state (vehicle alone; V) in each group. These measurements are confirmed in another independent set of experiments.

Airway hyper-responsiveness is a hallmark of allergic asthma [18]. To determine whether skin-derived TSLP could cause airway hyper-responsiveness in RBP-jCKO mice, we used the OVA-induced model of allergic inflammation and challenged the lung airways of age- and sex-matched RBP-jCKO;IL7rα-/-, RBP-jCKO, IL7rα-/-, and wild-type littermates twice with OVA. Thereafter, we measured the total lung resistance of OVA-treated mice at basal state (vehicle alone) or in response to increasing doses of nebulized methacholine. As expected, OVA-treated RBP-jCKO mice mounted a more severe airway resistance compared with their wild-type littermates when exposed to low doses of methacholine, and could not tolerate the higher doses of methacholine (Figure 4). RBP-jCKO;IL7rα-/-, however, did not develop any significant airway resistance even when exposed to high doses of methacholine (Figure 4). These results confirm that skin-barrier defects predispose animals to asthmatic phenotypes and that TSLP is required for this predisposition.

### TSLP Overexpression by the Skin Is Sufficient to Trigger the Atopic March

**Figure 5:**
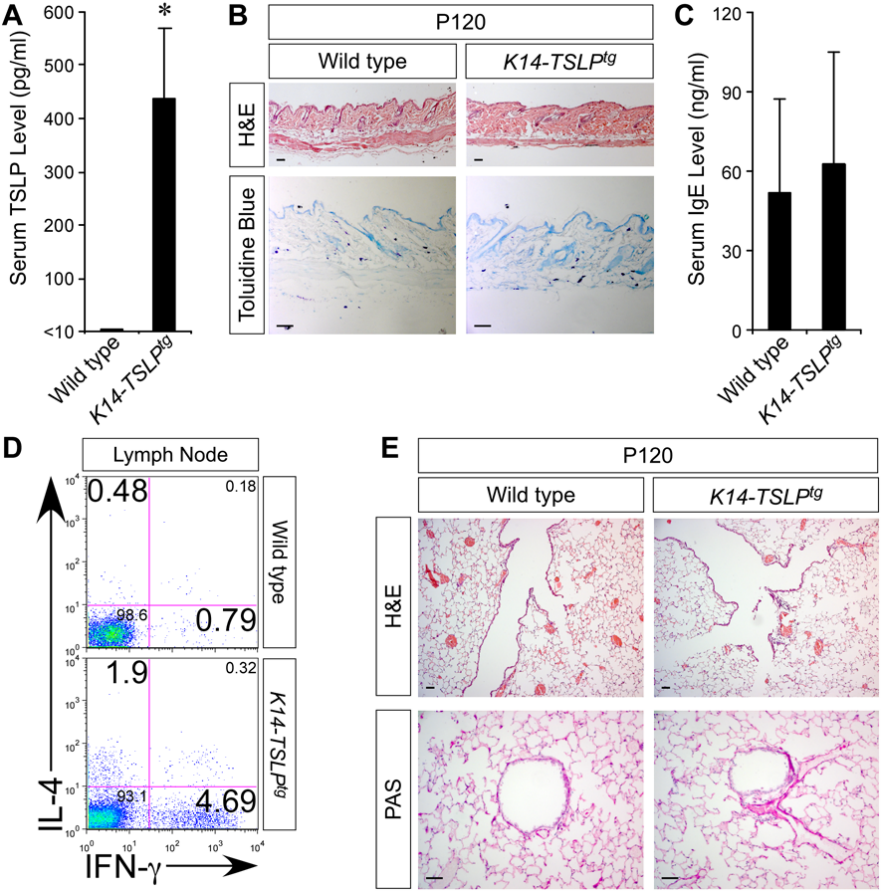
*K14-TSLP^tg^* mice exhibit no sign of skin or lung allergic inflammation under normal conditions. (A) Skin-derived TSLP overexpression leads to high systemic TSLP levels in the serum of *K14-TSLP^tg^* mice (*n* = 10 for each group; *p<0.01). (B–D) Under normal conditions, skin TSLP overproduction on an outbred C57BL/6-CD1 background does not elicit any allergic inflammation in *K14-TSLP^tg^* mice. Note the normal skin histology (B), normal serum IgE levels (*n* = 10 in each group)(C), and normal number of peripheral IL-4-producing Th2 cells (D) in *K14-TSLP^tg^* mice at postnatal day 120 (P120). (E) H&E and PAS stained lung sections of *K14-TSLP^tg^* animals confirm that no inflammation or airway remodeling occurred at P120 (scale bar: 50 µm).

Although we showed that TSLP signaling is necessary for the development of asthma in RBP-j–deficient animals with chronic allergic skin inflammation, it remains unclear whether other factors associated with AD-like skin lesions contribute to this atopic march. TSLP overexpression in mouse skin led to an AD-like pathology on an inbred background [26]. However, we discovered that on an outbred background, the same *K14-TSLP^tg^* mice [15] maintained high serum TSLP levels under normal conditions (approximately 450 pg/ml) without any skin or lung inflammation (Figure 5 and Figure S5) [29]. Normal skin morphology and the lack of mast cell hyperplasia evident in *K14-TSLP^tg^* mice confirmed that in this outbred background and in the absence of skin-barrier defects, TSLP overproduction was insufficient to attract mast cells to the skin (Figure 5B). In addition, serum IgE levels and Th2 cell numbers in the peripheral lymph nodes of *K14-TSLP^tg^* mice were indistinguishable from the wild type, confirming the absence of allergic skin inflammation in *K14-TSLP^tg^* animals that were not exposed to any external stimulus (e.g., allergen exposure; Figure 5C and 5D). Therefore, outbred *K14-TSLP^tg^* mice constituted a suitable model in which to examine whether epidermal TSLP overexpression alone would increase susceptibility to asthma upon allergen exposure.

**Figure 6:**
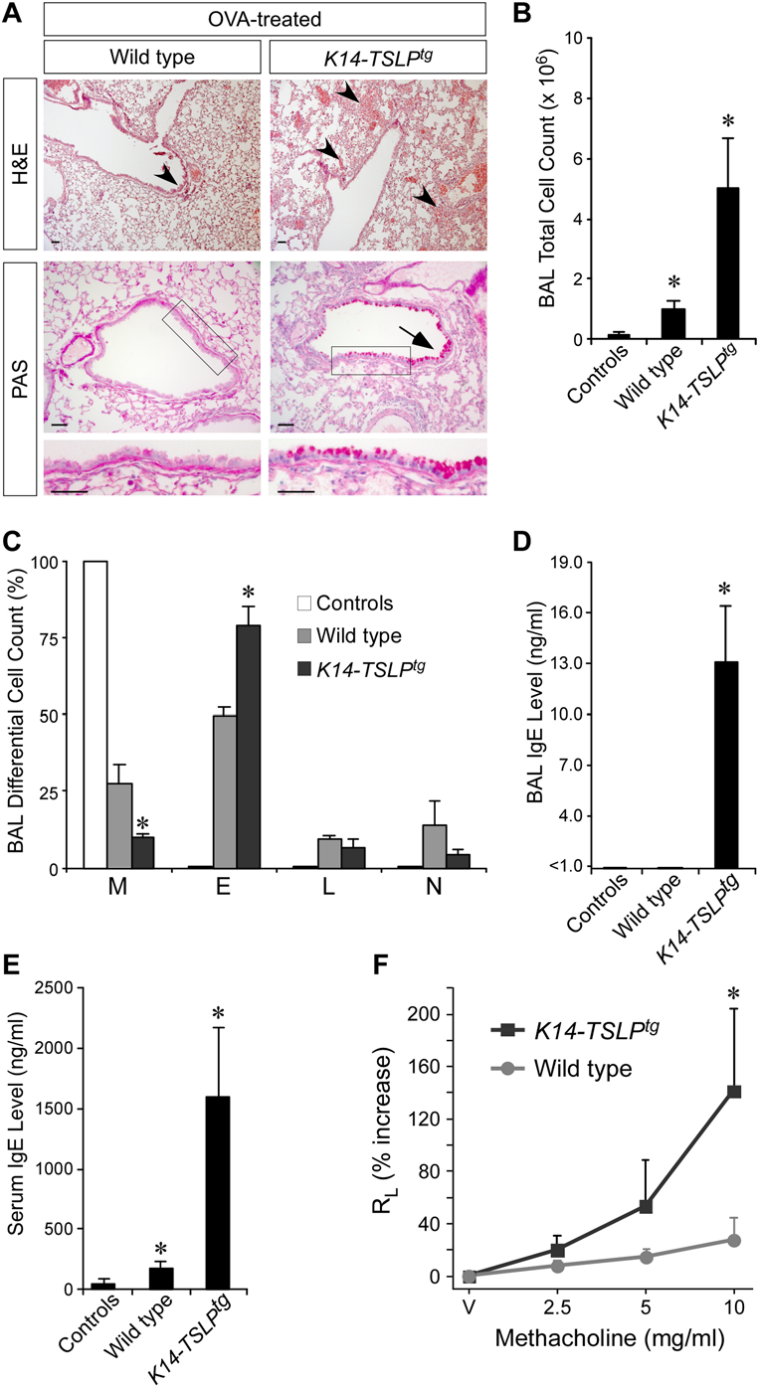
OVA treatment reveals that *K14-TSLP^tg^* mice are prone to asthmatic phenotype. (A) Histological analysis shows severe inflammatory cell infiltrates (arrowheads), airway remodeling, and goblet cell hyperplasia (arrow; insets) in the lung airways of 5- to 7-wk-old *K14-TSLP^tg^* animals that are sensitized and challenged with OVA, but not in similarly treated wild-type littermates (scale bar: 50 µm). (B–D) BAL fluid analysis shows more leukocytes (B), a higher percentage of eosinophils (C), and specific appearance of IgE in lung airways of OVA-treated *K14-TSLP^tg^* mice (D), confirming the higher intensity of the asthmatic phenotype conferred by the TSLP overexpression in the morphologically normal skin (M, macrophages; E, eosinophils; L, lymphocytes; N, neutrophils). (E) The severe allergic inflammation in OVA-treated *K14-TSLP^tg^* lungs leads to drastic elevation of serum IgE in the animals. “Controls” refers to *K14-TSLP^tg^* and wild-type mice treated with PBS alone (*n* = 4 for each group; *p<0.05, comparing the adjacent groups). (F) Total lung resistance (R_L_) measurements show that OVA-treated *K14-TSLP^tg^* lung airways are hyper-responsive to increasing doses of nebulized methacholine as compared to OVA-treated wild types (V: vehicle-only basal measurement; *n* = 4 for each group; *p<0.05). These findings are confirmed in additional independent experiments.

Next, we sensitized and challenged the lung airways of 5- to 7-wk-old *K14-TSLP^tg^* mice and their age- and sex-matched wild-type littermates three times with allergen. As seen in OVA-treated RBP-jCKO animals exposed to three doses of intranasal OVA, *K14-TSLP^tg^* mice developed a severe asthmatic response and significant lethality, which were not seen in the control group (Figure 6). Two out of eight OVA-treated transgenic mice died during the intranasal challenge. Histological examination of lungs from surviving *K14-TSLP^tg^* animals revealed pronounced airway remodeling with goblet cell hyperplasia and inflammation around the lung airways and vasculature (Figure 6A), which were negative for TSLP expression prior to OVA treatment (Figures S6 and S7). Total leukocyte count was less than 5-fold higher in BAL fluid from *K14-TSLP^tg^* mice compared with similarly treated wild-type animals (Figure 6B), which was mainly due to a severe BAL fluid eosinophilia in the transgenic animals (Figure 6C). In addition, IgE reached detectable levels only in BAL fluid from OVA-treated *K14-TSLP^tg^* mice (Figure 6D), which also experienced significantly higher serum IgE levels (Figure 6E) (1.6 µg/ml versus 0.18 µg/ml in OVA-treated wild-type serum, *p*<0.05). Thus, *K14-TSLP^tg^* mice developed an intense Th2 response upon allergen exposure. In the airway reactivity test, OVA-treated *K14-TSLP^tg^* mice showed a severe airway hyper-responsiveness to low doses of nebulized methacholine, which was not seen among similarly treated wild-type littermates (Figure 6F).

Even though outbred *K14-TSLP^tg^* animals developed a severe asthmatic phenotype, their skin retained its normal appearance after OVA treatment, confirming that the lung inflammation did not depend on a concurrent skin lesion (Figure S8). Although low levels of TSLP were expressed in the trachea, the *K14-TSLP* transgene was not expressed in the lung of *K14-TSLP^tg^* animals and TSLP protein was not detectable in their BAL fluid (Figures S6 and S7). Therefore, these observations are most consistent with a model in which systemic TSLP is sufficient to predispose mice to allergic lung inflammation.

## Discussion

This report describes the mechanism that mediates the progression of a local allergic skin disease to a disseminated allergic inflammation downstream of skin-barrier defects in mice. We show that creating an intrinsic skin-barrier defect (by removing Notch signaling specifically from some skin keratinocytes) results in the sensitization of the lung airways to allergens. Similar to the clinical cases, barrier-defective mice require a secondary insult by allergens to expose their predisposition to asthma. Importantly, we show that epicutaneous sensitization with a common allergen [3] is not required to achieve this pronounced lung hyper-responsiveness. Therefore, RBP-jCKO animals demonstrate that skin-barrier impairment provides a signal that promotes development of a distant atopy in the lung, most likely by a systemic, diffusible cytokine. We identified this signal to be TSLP, a central player in the early stages of allergic inflammation [18]–[20] and a barrier-defect–sensitive product of skin keratinocytes that is released prior to development of an AD-like skin lesion [15]. In contrast to TSLP overexpression by lung epithelia, which does not result in systemic accumulation of this cytokine [18], overexpression of TSLP in the skin results in high systemic availability [15]. Therefore, skin is capable of acting as a signaling organ, driving susceptibility to allergic inflammation in another barrier organ (i.e., lung) by releasing TSLP.

Importantly, we were able to show that TSLP was necessary to predispose RBP-j–deficient animals to the asthmatic phenotype. Concomitant removal of IL7Rα subunit of the TSLP receptor in RBP-jCKO mice prevented the atopic march despite persistent AD-like pathology and elevated serum IgE levels. Complementing this experiment is the observation that overexpression of TSLP by skin keratinocytes leads to high serum levels of TSLP that are sufficient to sensitize the lung airways to allergic inflammation in the absence of any skin pathology. If the low levels of TSLP in the trachea of *K14-TSLP^tg^* mice contribute to the phenotype, they do so by adding to the already high levels of skin-derived serum TSLP. Because TSLP can activate dendritic cells [30], T cells [31],[32], and myeloid cells [33], we speculate that its high systemic levels directly prime these immune cells at distant sites (e.g., the lung) to mount an intense allergic inflammation in response to a second stimulatory signal (e.g., allergens in the lung airways); however, the specific contribution of these immune cells to the atopic march downstream of TSLP-mediated activation remains a subject for future investigation. Of note, elevated TSLP expression is also reported in psoriatic skin [34]; however, there is no evidence linking psoriasis to predisposition to asthma. This phenomenon could be explained by the dominance of Th1 response in patients with psoriasis and the unresponsiveness of their immune cells to TSLP [34].

The findings outlined in this report have a potentially important implication for human health. They provide a plausible explanation of why a large number of AD patients develop asthma and other allergic disorders later in life [35],[36]. Because TSLP overproduction is triggered by skin-barrier defects and faithfully mirrors the severity of these defects [15], our data emphasize that an early and aggressive treatment of the underlying skin-barrier defects in AD-prone patients [10],[11] may be more beneficial in preventing asthma than treating the outbreaks of AD lesions [6]. Although serum TSLP levels in AD patients are yet to be determined, high TSLP expression levels in human AD lesions [37] suggest that this diffusible cytokine could also reach systemic levels sufficient to trigger atopic march in AD patients. If indeed serum TSLP levels are elevated in AD patients, our findings suggest that an aggressive management of TSLP levels in these patients will lower the incidence of asthma later in their lives.

## Materials and Methods

### Mice

*Msx2-Cre/+;RBP-j^flox/flox^* (RBP-jCKO), *Msx2-Cre/+;RBP-j^flox/flox^; IL7r*α-/- (RBP-jCKO;IL7rα-/-), and *K14-TSLP* ^*tg*^ mice were generated as previously described [15]. All animals were kept in mixed C57BL/6 and CD1 outbred genetic background, which are resistant to Th2-mediated inflammation [29]. Age- and sex-matched mutant and wild-type littermates were used in each analysis. All mice were maintained in the animal facility under Washington University animal care regulations.

### OVA Treatment

The allergic sensitization and lung airways challenge with OVA was carried out as previously outlined [18]. In brief, wild-type and mutant mice at 5–7 wk of age were sensitized on days 0 and 14 by 250 µl intraperitoneal injection of antigen solution containing 50 µg OVA (Sigma) dissolved in 1.3 mg aluminum hydroxide gel (Sigma) and PBS. On days 21, 22, and 23, mice were intranasally challenged with 150 µg OVA dissolved in 40 µl of PBS. Mice cohorts designated as “controls” included the mutant and wild-type animals that underwent the same regimen without OVA antigen. On day 24, all the animals were humanely euthanized for the analysis. To avoid mortality among RBP-jCKO animals, all the age- and sex-matched littermates used in the rescue experiments presented in Figures 3 and 4 were challenged only twice with OVA. Note that RBP-jCKO;IL7rα-/- mice could tolerate the repeated OVA intranasal challenge, but RBP-jCKO mice developed severe responses after even two exposures. The OVA experiments were conducted on male and female animals; however, within-sex analyses were performed to avoid any sex effect on phenotypes observed.

### Airway Reactivity Test

The airway responsiveness to aerosolized methacholine was determined in OVA-treated animals by measuring total lung resistance and dynamic compliance as previously outlined [38]. In brief, mice were treated with OVA as described in the preceding section (two intranasal OVA challenges for all mice in rescue experiments (Figure 4) and three intranasal OVA challenges for all mice in sufficiency experiments (*K14-TSLP^tg^* and wild type; Figure 6)). The animals were then anesthetized for airway reactivity test on day 24 post OVA sensitization. They were ventilated through a tracheotomy and monitored for intrapleural pressure using an oroesophageal tube. PBS (vehicle) or methacholine (Sigma) in PBS were delivered at 3-min intervals using an in-line nebulizer. The respiratory flow signals were collected between deliveries using a pneumotach (SenSym SCXL004, Buxco Electronics). For detailed description of the device, refer to [39].

### Histology

Dorsal skin samples were harvested from the mice and fixed in 4% PFA at 4 °C overnight. Lungs were inflated through the trachea to 25-cm water pressure with 4% PFA prior to excision from the chest and fixation. These lungs were PFA-fixed for 24 hr at 4 °C and embedded in paraffin. The paraffin-embedded tissues were sectioned at 5–6 µm and stained with hematoxylin and eosin (H&E), toluidine blue, or periodic acid-Schiff (PAS). For RBP-j immunohistochemical staining, anti–RBP-j antibody (clone T6709, Institute of Immunology) and biotinylated anti–rat secondary antibody were used. HRP-conjugated streptavidin and DAB substrate kit (Pierce) were used to visualize the signal. Hematoxylin was used to counterstain the sections. For TSLP immunostaining, paraffin-embedded tissue samples and biotinylated anti-TSLP antibody (R&D Systems) were used. Sections were counterstained with DAPI nuclear stain.

### BAL Analyses

BAL fluid was collected by infusing the lungs of the anesthetized mouse with 1-ml PBS through tracheal insertion of a Surflo catheter (Terumo Medical). Leukocyte count in BAL fluid was determined using a Hemavet 950 analyzer (Drew Scientific). BAL fluid was spun down and the supernatant was stored for cytokine/immunoglobulin analysis. The cell pellet was resuspended in PBS and used for differential cell count after Giemsa staining on the slide.

### ELISA

Serum TSLP levels were determined using Quantikine mouse TSLP kit according to manufacturer's instructions (R&D Systems). Serum and BAL fluid IgE levels were measured using Mouse IgE ELISA kit (Immunology Consultants Laboratory).

### Flow Cytometry

Intracellular cytokine staining was conducted to estimate Th2 cell census as previously described [26]. Single cell suspensions were prepared from spleen and lymph node samples and cultured in presence of PMA (50 ng/ml), ionomycin (1 µg/ml) and monensin (10 µg/ml) for 4 h. Cells were then stained with phycoerythrin (PE)-cy7 conjugated anti-CD4 antibody (552775, BD Bioscience Pharmingen). After fixation in 2% PFA and permeabilization with 0.5% saponin, cells were stained with PE conjugated anti-IL4 (554389) and APC conjugated anti-IFN-γ (554412) antibodies from BD Bioscience Pharmingen [40].

### PCR

Conventional PCR for the *RBP-j* allele was performed on genomic DNA isolated from skin and lung of adult RBP-jCKO mice using KlenTaq10 (DNA Polymerase Technology) supplemented with 1.3 M final concentration of betaine (amplification cycles = 32). The following primers were used to distinguish between deleted (*RBP-j^Δ^*) and floxed (*RBP-j^flox^*) alleles of *RBP-j*: Deleted allele: 5′-TGTTTGCCACCAGAATCTGTTTGTTATTTGC-3′ and 5′-ATTTGCTTGAGGCTTGATGTTCTGTATTGC-3′.

Floxed allele: 5′-TGTTTGCCACCAGAATCTGTTTGTTATTTGC-3′ and 5′-AGGTACCTGGTACTAACTGTCTGGGACCG-3′.

### qRT-PCR

mRNA isolated from P4 epidermis and lung of RBP-jCKO and wild-type littermates was used to perform qRT-PCR analysis as previously described [41]. The primers used to amplify *TSLP* were: 5′-CCAGGCTACCCTGAAACTGA-3′ and 5′-TCTGGAGATTGCATGAAGGA-3′.

### Statistical Analysis

The quantitative measurements were assessed using Student *t-*test as the test of significance and presented as mean ± standard deviation in bar graph format. Our studies were conducted in outbred cohorts of animals; therefore, we used nested ANOVA to exclude any effect of between-family differences and make certain the significant differences observed were solely attributable to the gene removed (RBP-jCKO and RBP-jCKO;IL7rα-/-) or overexpressed (*K14-TSLP^tg^*).

### Accession Numbers

The Genbank (http://www.ncbi.nlm.nih.gov/Genbank/) accession numbers for proteins discussed in this article are as follows: RBP-j, NM_009035; TSLP, NM_021367.

## Supporting Information

Figure S1 RBP-jCKO animals die prematurely due to their severe skin phenotype (*p*<0.001 compared to wild-type life span, log-rank test). A few mutant mice that survive up to one year (red circles), however, develop spontaneous lung inflammation.
(78 KB TIF)

Figure S2 Serum TSLP levels in RBP-jCKO mice are highly elevated. TSLP overproduction is evident in RBP-jCKO serum at 1 wk after birth, reaching extreme levels in the adult animals (*n* = 4 for each group; *p<0.01, comparing the mutants to the wild-type littermates).
(55 KB TIF)

Figure S3 Lung epithelium is normal in RBP-jCKO animals. (A) PCR analysis of DNA isolated from adult RBP-jCKO (*Msx2-Cre/+;RBP-j^flox/flox^*) and wild-type (*RBP-j^flox/flox^*) skin and lung shows that *RBP-j* locus is intact (i.e., *RBP-j* is not deleted) in the lung (Δ: deleted allele; M: molecular marker; S: skin; L: lung). (B) Immunohistochemical analysis for RBP-j protein confirms that RBP-j is present in the lung airway epithelium. Skin sections stained under the same condition are presented as controls (scale bar: 50 µm).
(7.35 MB TIF)

Figure S4 Serum TSLP levels of 10-wk-old RBP-jCKO;IL7rα-/- mice are highly elevated. This indicates that skin-barrier defects caused by the loss of *RBP-j* in epidermal keratinocytes persist in the absence of *IL7R*α (*n* = 5 for each group; * *p*<0.01). Note that serum TSLP levels in RBP-jCKO;IL7rα-/- mice are consistently and significantly higher than in RBP-jCKO animals. We are currently investigating the underlying reason for this surge.
(50 KB TIF)

Figure S5 The ear and skin of *K14-TSLP^tg^* mice appear normal at P120. This emphasizes that in an outbred genetic background (C57BL/6 and CD1 mix) these transgenic animals do not develop any skin inflammation under normal conditions.
(1.3 MB TIF)

Figure S6 There is no TSLP overexpression detectable in *K14-TSLP^tg^* lung airways or parenchyma. Because the *K14* gene is expressed in basal cells located in the trachea, we analyzed mRNA levels by qRT-PCR on samples isolated from epidermis, trachea and lung of *K14-TSLP^tg^* and wild-type mice. This analysis shows that the *K14-TSLP* transgene is active in basal cells within the trachea, but the overall levels are 200-fold lower than those made by epidermal keratinocytes (***p<0.0000001 and *p<0.01 compared to wild type).
(145 KB TIF)

Figure S7 Lung epithelium does not overexpress TSLP in adult *K14-TSLP^tg^*, RBP-jCKO, or RBP-jCKO;IL7rα-/- animals. Immunofluorescence staining for TSLP protein (red) confirms that TSLP is overexpressed only in the epidermal keratinocytes of the mutant mice. All sections are stained under the same conditions. Dotted lines outline the basement membrane and asterisks highlight the epidermal keratin cysts present in RBP-jCKO skin (scale bar: 50 µm).
(5.3 MB TIF)

Figure S8 The skin of OVA-treated *K14-TSLP^tg^* mice remains normal. H&E and toluidine blue staining of *K14-TSLP^tg^* and wild-type skin show no significant signs of cutaneous inflammation in the transgenic mice (scale bar: 50 µm).
(1.3 MB TIF)

Table S1 Major antimicrobial peptides are overexpressed in postnatal epidermis of RBP-jCKO animals. This overexpression indicates the persistence of the skin-barrier defect in RBP-jCKO mice after birth [25]. Data are extracted from a microarray study on P9 epidermis of RBP-jCKO and wild-type littermates as previously described [15]. The fold increase of antimicrobial peptide mRNA in RBP-j–deficient epidermis relative to wild type is presented.
(37 KB PDF)

## Abbreviations

AD: atopic dermatitis
BAL: bronchoalveolar lavage
IL: interleukin
OVA: ovalbumin
Th2: T-helper2
TSLP: thymic stromal lymphopoietin

## Note Added in Proof

After this manuscript was accepted for publication, similar observations were reported by Zhang et al: Zhang Z, Hener P, Frossard N, Kato S, Metzger D, et al. (2009). Thymic stromal lymphopoietin overproduced by keratinocytes in mouse skin aggravates experimental asthma. Proc Natl Acad Sci U S A 106: 1536–1541.
