# Supplementary figures and images for: Skin-Derived TSLP Triggers Progression from Epidermal-Barrier Defects to Asthma
Source: PLoS Biol. 2009 May 19;7(5):e1000067. doi: 10.1371/journal.pbio.1000067 (PMC2700555; doi:10.1371/journal.pbio.1000067)

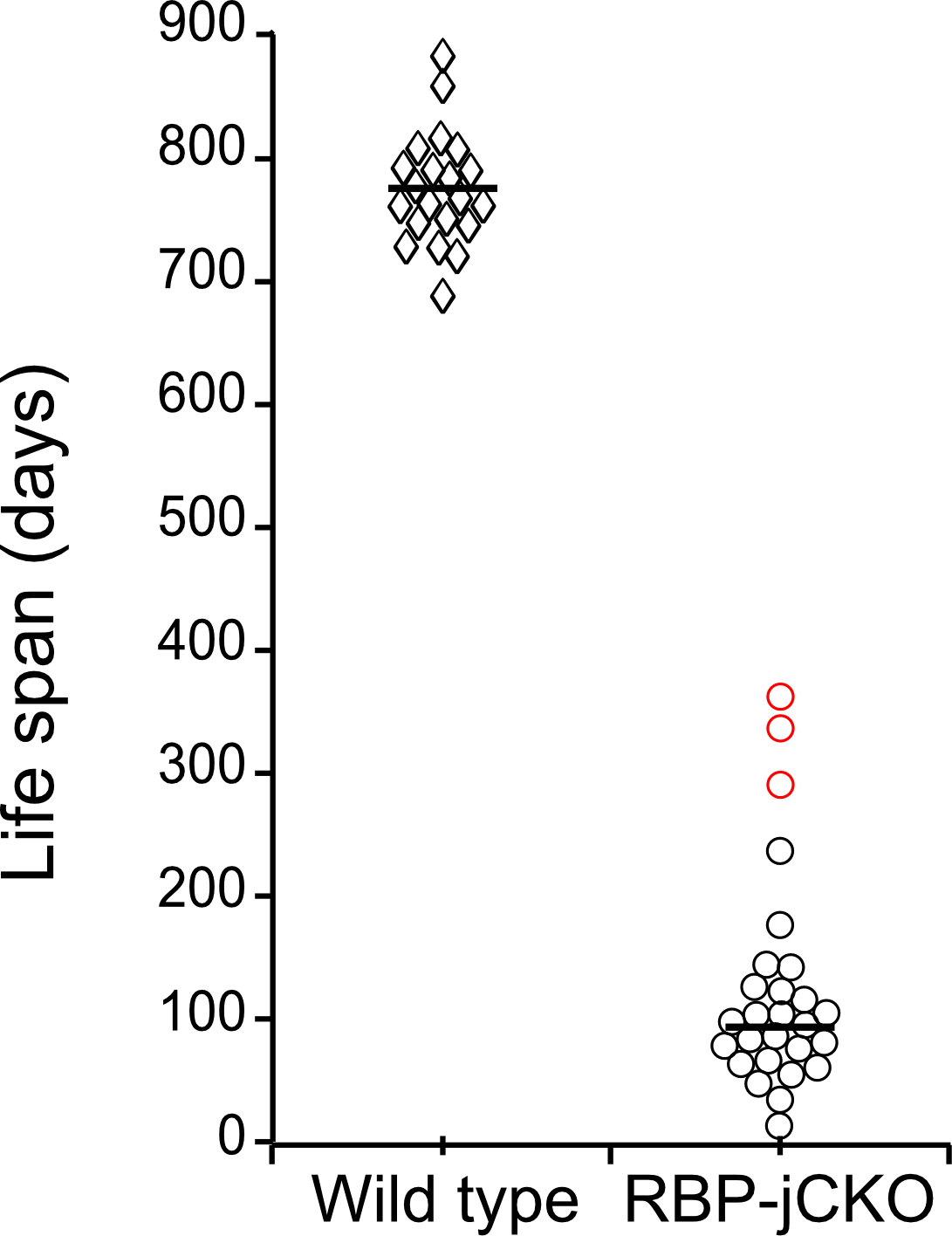

Supplement: Figure S1 — RBP-jCKO animals die prematurely due to their severe skin phenotype (p<0.001 compared to wild-type life span, log-rank test). A few mutant mice that survive up to one year (red circles), however, develop spontaneous lung inflammation. (78 KB TIF) [file pbio.1000067.s001.tif]

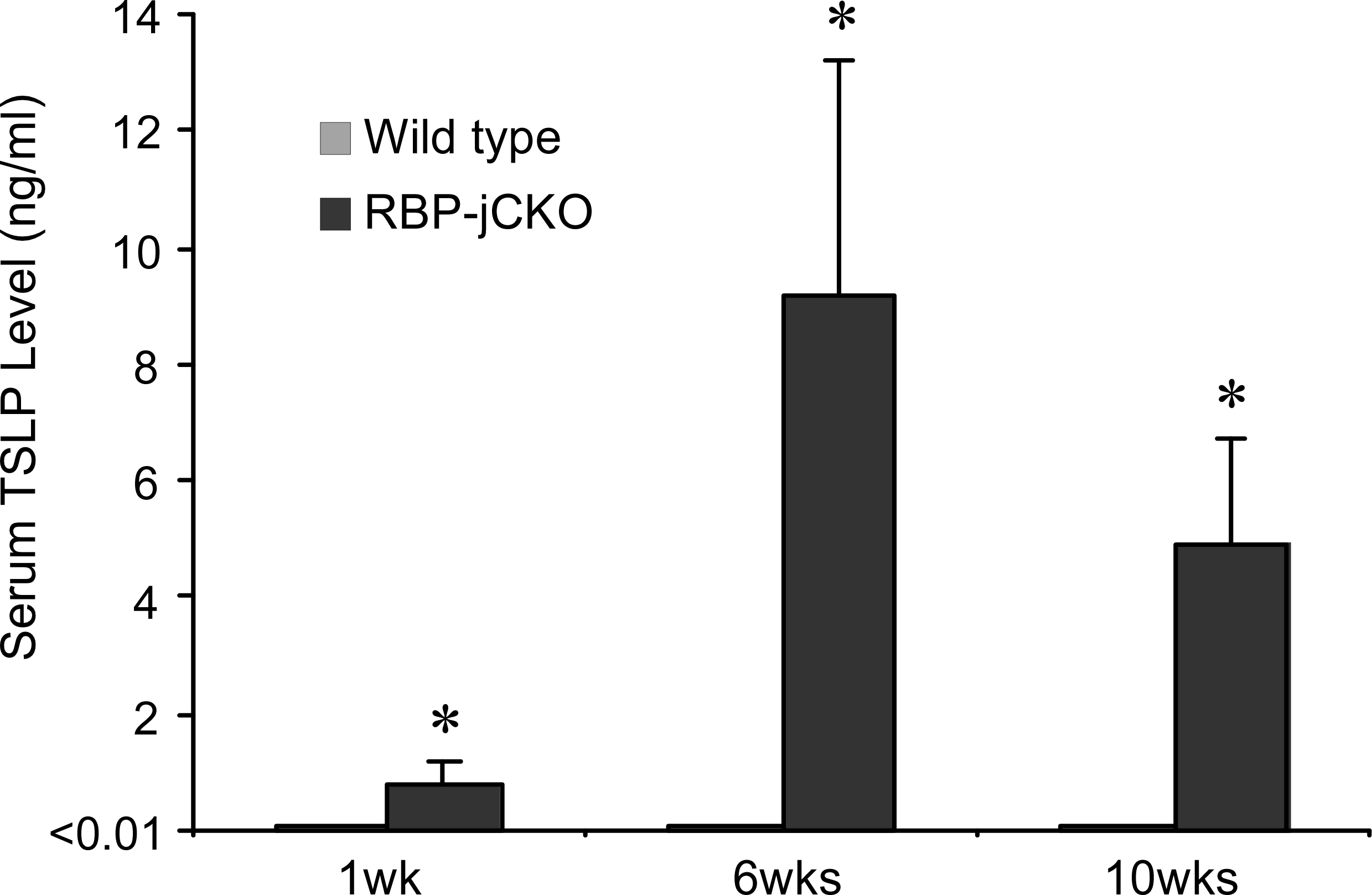

Supplement: Figure S2 — Serum TSLP levels in RBP-jCKO mice are highly elevated. TSLP overproduction is evident in RBP-jCKO serum at 1 wk after birth, reaching extreme levels in the adult animals (n = 4 for each group; *p<0.01, comparing the mutants to the wild-type littermates). (55 KB TIF) [file pbio.1000067.s002.tif]

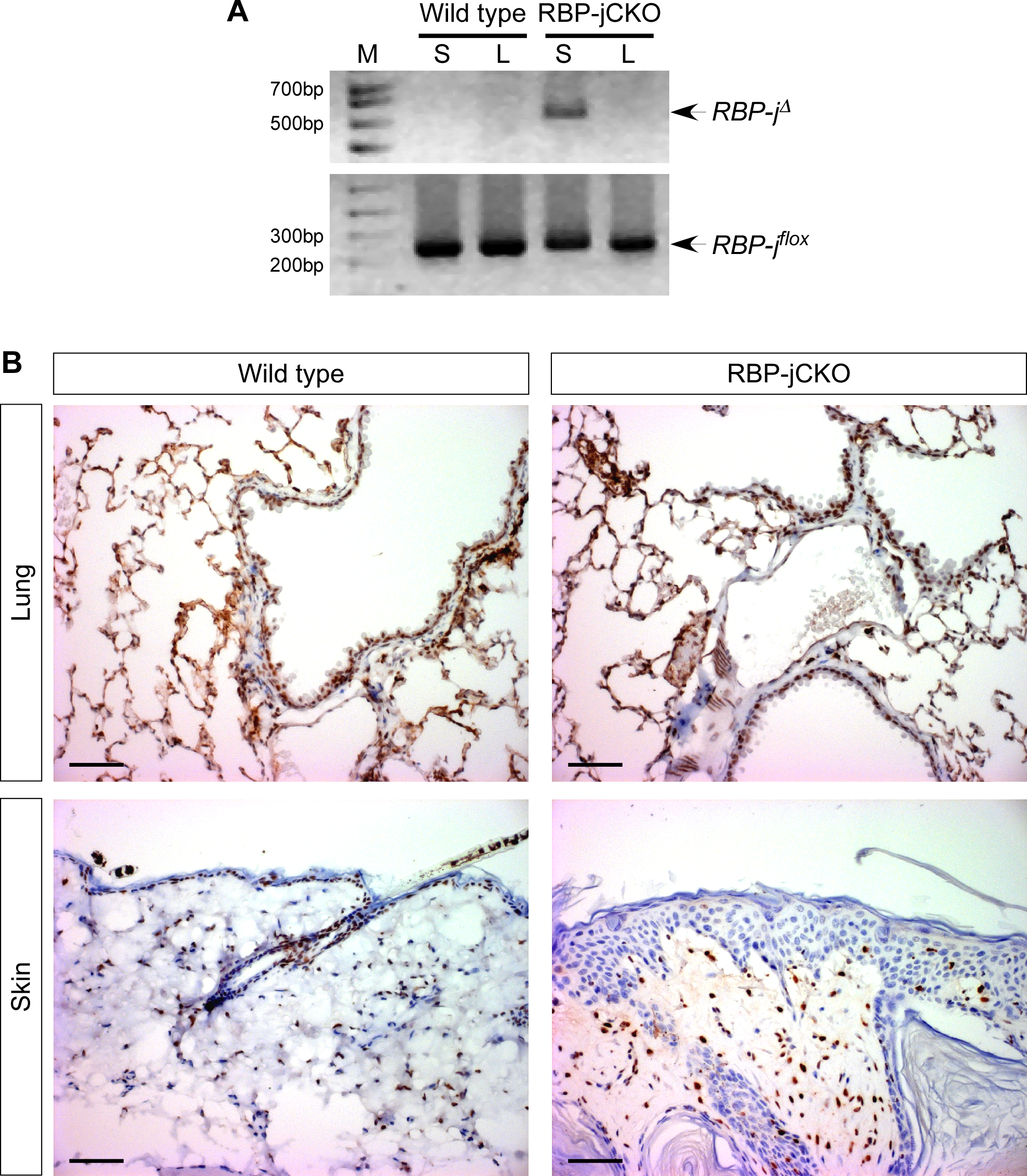

Supplement: Figure S3 — Lung epithelium is normal in RBP-jCKO animals. (A) PCR analysis of DNA isolated from adult RBP-jCKO (Msx2-Cre/+;RBP-jflox/flox) and wild-type (RBP-jflox/flox) skin and lung shows that RBP-j locus is intact (i.e., RBP-j is not deleted) in the lung (Δ: deleted allele; M: molecular marker; S: skin; L: lung). (B) Immunohistochemical analysis for RBP-j protein confirms that RBP-j is present in the lung airway epithelium. Skin sections stained under the same condition are presented as controls (scale bar: 50 µm). (7.35 MB TIF) [file pbio.1000067.s003.tif]

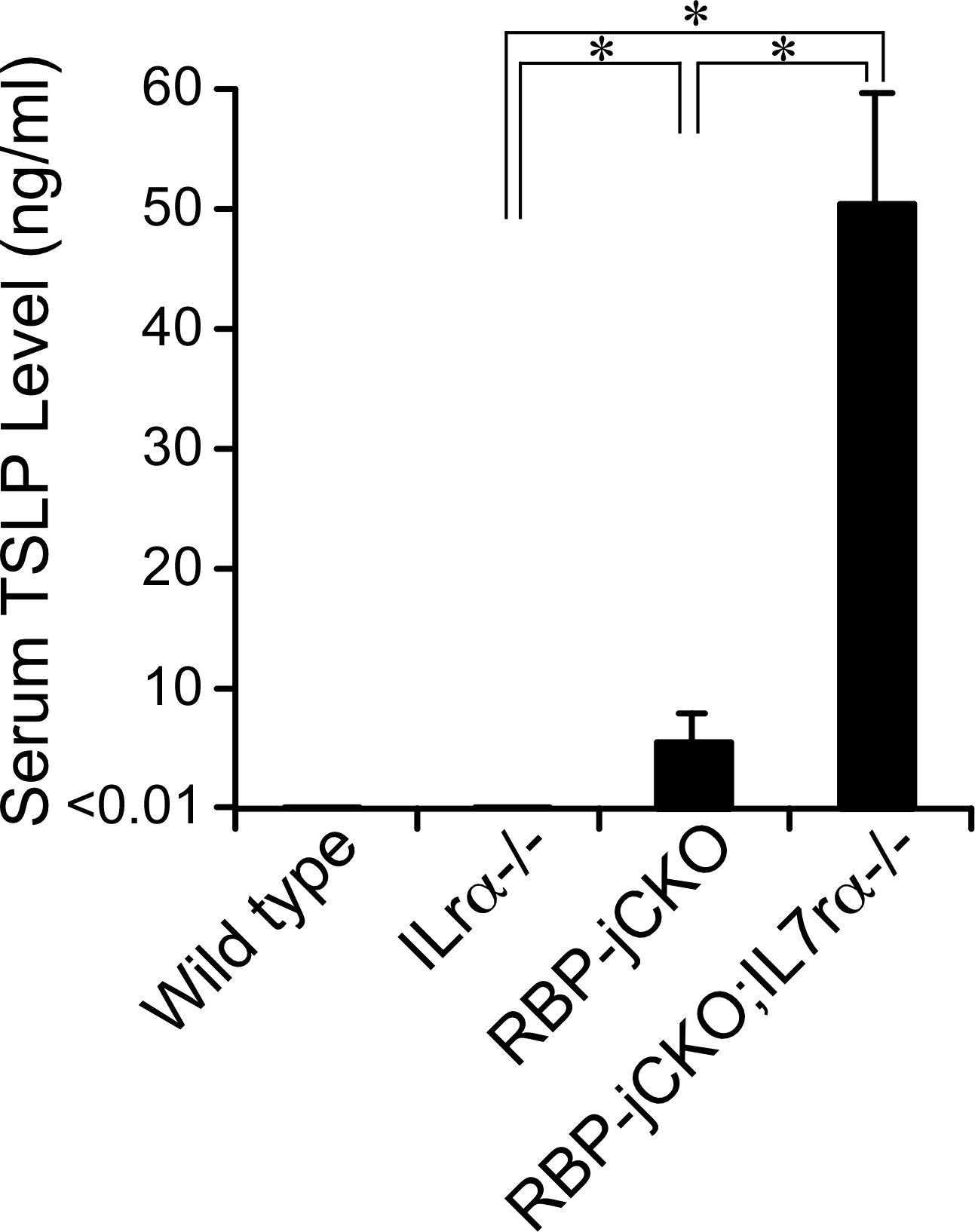

Supplement: Figure S4 — Serum TSLP levels of 10-wk-old RBP-jCKO;IL7rα-/- mice are highly elevated. This indicates that skin-barrier defects caused by the loss of RBP-j in epidermal keratinocytes persist in the absence of IL7Rα (n = 5 for each group; * p<0.01). Note that serum TSLP levels in RBP-jCKO;IL7rα-/- mice are consistently and significantly higher than in RBP-jCKO animals. We are currently investigating the underlying reason for this surge. (50 KB TIF) [file pbio.1000067.s004.tif]

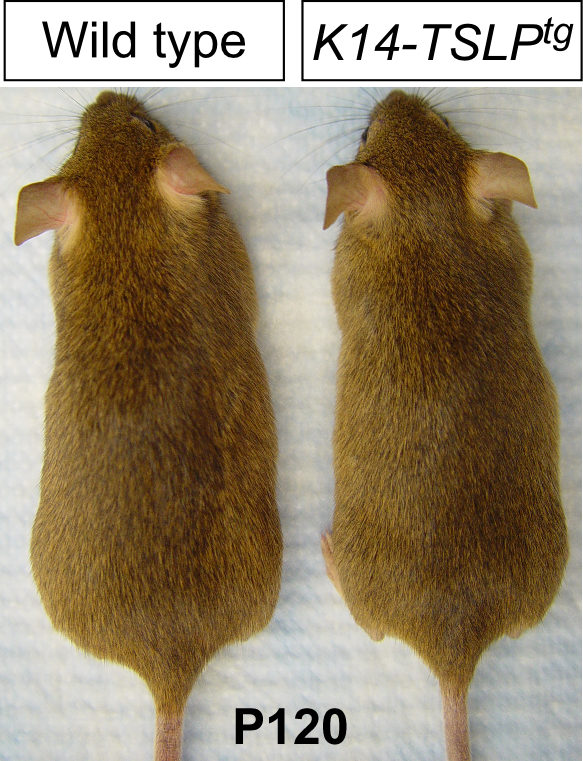

Supplement: Figure S5 — The ear and skin of K14-TSLPtg mice appear normal at P120. This emphasizes that in an outbred genetic background (C57BL/6 and CD1 mix) these transgenic animals do not develop any skin inflammation under normal conditions. (1.3 MB TIF) [file pbio.1000067.s005.tif]

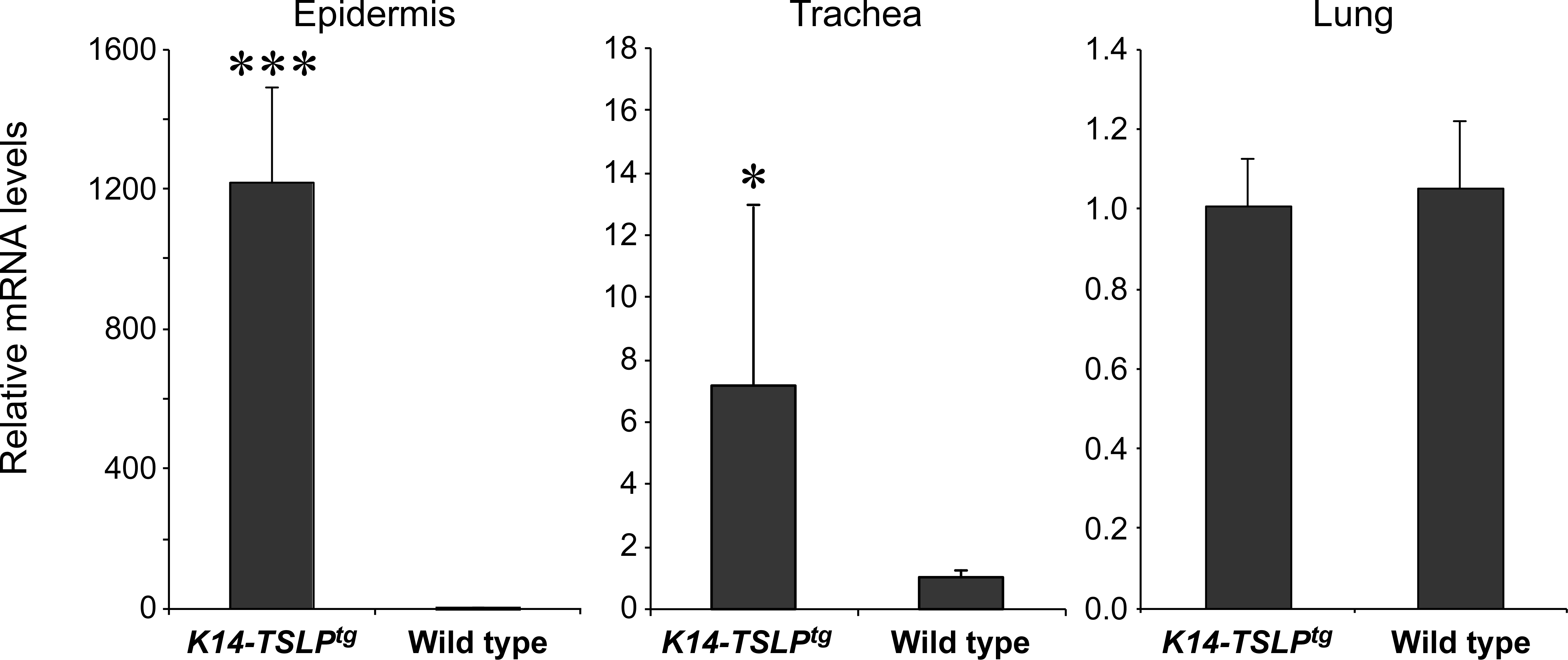

Supplement: Figure S6 — There is no TSLP overexpression detectable in K14-TSLPtg lung airways or parenchyma. Because the K14 gene is expressed in basal cells located in the trachea, we analyzed mRNA levels by qRT-PCR on samples isolated from epidermis, trachea and lung of K14-TSLPtg and wild-type mice. This analysis shows that the K14-TSLP transgene is active in basal cells within the trachea, but the overall levels are 200-fold lower than those made by epidermal keratinocytes (***p<0.0000001 and *p<0.01 compared to wild type). (145 KB TIF) [file pbio.1000067.s006.tif]

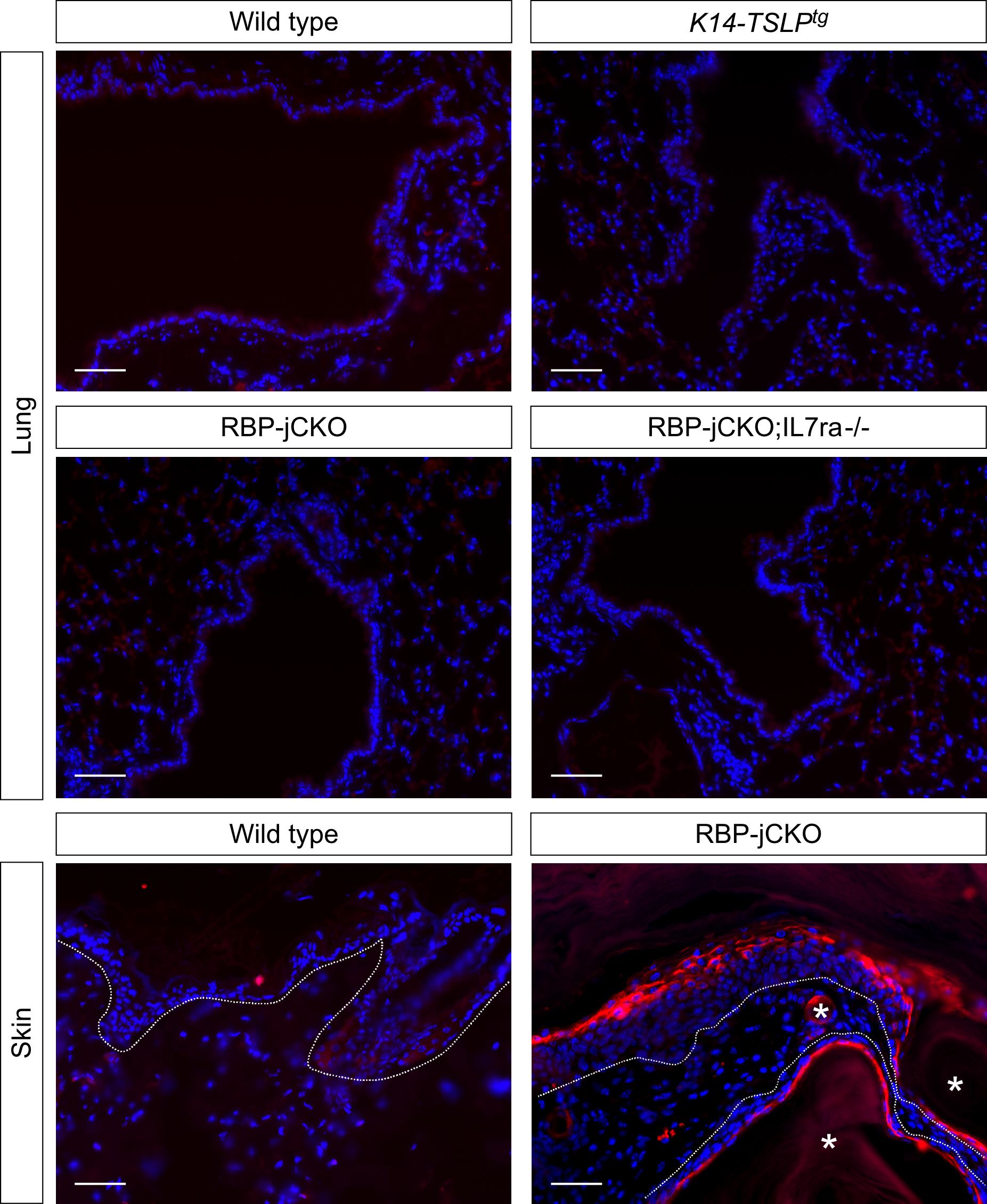

Supplement: Figure S7 — Lung epithelium does not overexpress TSLP in adult K14-TSLPtg, RBP-jCKO, or RBP-jCKO;IL7rα-/- animals. Immunofluorescence staining for TSLP protein (red) confirms that TSLP is overexpressed only in the epidermal keratinocytes of the mutant mice. All sections are stained under the same conditions. Dotted lines outline the basement membrane and asterisks highlight the epidermal keratin cysts present in RBP-jCKO skin (scale bar: 50 µm). (5.3 MB TIF) [file pbio.1000067.s007.tif]

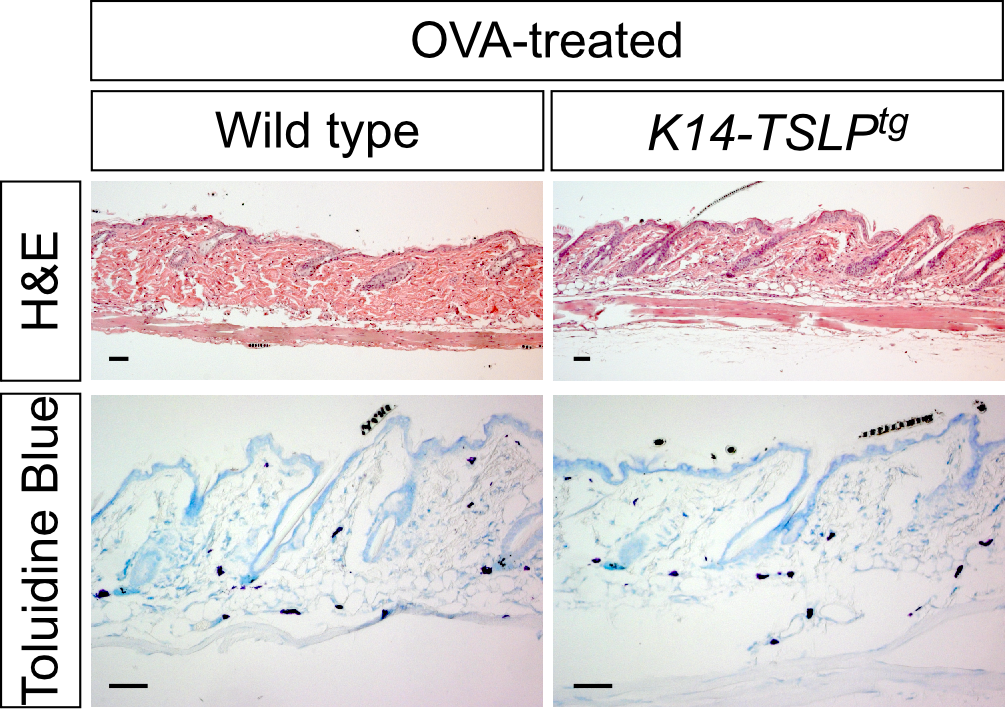

Supplement: Figure S8 — The skin of OVA-treated K14-TSLPtg mice remains normal. H&E and toluidine blue staining of K14-TSLPtg and wild-type skin show no significant signs of cutaneous inflammation in the transgenic mice (scale bar: 50 µm). (1.3 MB TIF) [file pbio.1000067.s008.tif]
