# Supplementary material for: Skin-Derived TSLP Triggers Progression from Epidermal-Barrier Defects to Asthma
Source: PLoS Biol. 2009 May 19;7(5):e1000067. doi: 10.1371/journal.pbio.1000067 (PMC2700555; doi:10.1371/journal.pbio.1000067)
Supplement: Table S1 — Major antimicrobial peptides are overexpressed in postnatal epidermis of RBP-jCKO animals. This overexpression indicates the persistence of the skin-barrier defect in RBP-jCKO mice after birth [25]. Data are extracted from a microarray study on P9 epidermis of RBP-jCKO and wild-type littermates as previously described [15]. The fold increase of antimicrobial peptide mRNA in RBP-j–deficient epidermis relative to wild type is presented. (37 KB PDF) [file pbio.1000067.s009.pdf]

**Table S1.**

| Gene Symbol | Gene Title | Fold Increase (RBP-jCKO/Wt) |
|---|---|---|
| **S100a8** | **Mus musculus S100 calcium binding protein A8 (calgranulin A)** | **16.454** |
| **S100a9** | **Mus musculus S100 calcium binding protein A9 (calgranulin B)** | **12.240** |
| **Defb3** | **Mus musculus defensin beta 3 (Defb3)** | **2.928** |
